# Manzamine-A Alters In Vitro Calvarial Osteoblast Function

**DOI:** 10.3390/md20100647

**Published:** 2022-10-19

**Authors:** Samantha Hardy, Yeun-Mun Choo, Mark Hamann, James Cray

**Affiliations:** 1 Department of Biomedical Education and Anatomy, College of Medicine, The Ohio State University, Columbus, OH 43210, USA; 2 Chemistry Department, Faculty of Science, University of Malaya, Kuala Lumpur 50603, Malaysia; 3 Departments of Drug Discovery and Biomedical Sciences and Public Health, Colleges of Pharmacy and Medicine, Medical University of South Carolina, Charleston, SC 29425, USA; 4 Division of Biosciences, The Ohio State College of Dentistry, Columbus, OH 43210, USA

**Keywords:** manzamine, bone, osteoblast, six1, osteogenesis

## Abstract

Manzamine-A is a marine-derived alkaloid which has anti-viral and anti-proliferative properties and is currently being investigated for its efficacy in the treatment of certain viruses (malaria, herpes, HIV-1) and cancers (breast, cervical, colorectal). Manzamine-A has been found to exert effects via modulation of SIX1 gene expression, a gene critical to craniofacial development via the WNT, NOTCH, and PI3K/AKT pathways. To date little work has focused on Manzamine-A and how its use may affect bone. We hypothesize that Manzamine-A, through SIX1, alters bone cell activity. Here, we assessed the effects of Manzamine-A on cells that are responsible for the generation of bone, pre-osteoblasts and osteoblasts. PCR, qrtPCR, MTS cell viability, Caspase 3/7, and functional assays were used to test the effects of Manzamine-A on these cells. Our data suggests Six1 is highly expressed in osteoblasts and their progenitors. Further, osteoblast progenitors and osteoblasts exhibit great sensitivity to Manzamine-A treatment exhibited by a significant decrease in cell viability, increase in cellular apoptosis, and decrease in alkaline phosphatase activity. In silico binding experiment showed that manzamine A potential as an inhibitor of cell proliferation and survival proteins, i.e., Iκb, JAK2, AKT, PKC, FAK, and Bcl-2. Overall, our data suggests Manzamine-A may have great effects on bone health overall and may disrupt skeletal development, homeostasis, and repair.

## 1. Introduction

Appropriate preclinical and clinical screening for pharmacological therapies is necessary to inform the patient populations of interest which may benefit from these therapies but could incur risks associated with exposure to their health [1,2,3]. The United States Food and Drug Administration and the Center for Drug Evaluation and Research is the clearing house for this information garnered from a variety of sources (academic, pharmaceutical companies, NIH) using label approaches that inform specific de novo effects and those associated with existing comorbidities [1,2,4,5,6]. Note many drugs authorized for emergency usage, currently in clinical trials, or used in off-label contexts may not have adequate risk/benefit assessment for those patients who are being treated. This is especially poignant today as the current COVID crisis has altered our focus on emerging disease and has driven an increase in emergency authorizations and off-label use of many drugs as tools to contain emerging viruses but can also adversely affect vulnerable populations [7,8,9,10,11,12,13,14,15,16,17,18].

Marine natural products remain a highly important source for emerging drugs, especially in the treatments of cancers and viral infections [19,20,21,22,23,24,25,26]. One such potential drug is manzamine-A, a marine sponge derived alkaloid that emerged in the field of infectious disease as a promising control for malaria [27,28,29,30,31,32,33,34,35]. The understanding of manzamine and derived analogs utility in medicine is nascent, but is proposed to include treatment for malaria [27,28,29,30,31,32,33], herpes [29,36,37,38], HIV [29,32,39,40,41,42,43,44], cancer [6,10,13,35,37,38,39,44,45], as well as generally having anti-bacterial, antifungal, and anti-inflammatory activities [15,17,19,20,24,32,34,36,37,38,43,46,47,48,49,50,51,52,53,54,55]. Thus, this emerging drug may show great potential in the treatment of many diseases.

One mechanism of action by which manzamine-A acts is as a novel small molecule inhibitor targeting cells that express a critical homeobox gene *SIX1* [45]. *SIX1* expression has been linked to organogenesis, DNA specificity, protein–protein interactions, and proliferation and survival of cells [45,46,47,48,49,50,51,52,53,54,55,56,57,58,59,60,61,62,63,64,65,66,67,68,69,70,71,72,73,74,75,76,77,78,79,80,81,82,83,84,85]. Thus, from a cellular development and maintenance perspective the use of manzamine-A and related alkaloid analogs may be of concern. Strengthening this argument are data from the murine knockout model that present with multiple anomalies including those related to the musculoskeletal system [61,66]. *SIX1* has also been shown to segregate in birth defects related to bone development leading to excess bone formation [86]. Importantly, if manzamine-A use is suspected of affecting bone health, additional patient populations may be implicated including those susceptible to bone loss diseases. The US-CDC reports the age-adjusted prevalence of osteoporosis has increased in the last decade 9.5% in 2008 to 12.6% in 2018 [87]. Furthermore, the prevalence of osteoporosis leads to a significant increased risk of related bone fractures leading to morbidity and mortality and even death (33% of hip fractures in patients over 50 die within a year) [88,89,90,91,92]. Thus, a better understanding of “if and how” manzamine-A interacts with the cell population responsible for bone development and remodeling, the osteoblast, and the predicted target Six-1 is now necessary.

Here, we have taken that first step to characterize if manzamine-A affects osteoblasts, the cells responsible for mineralizing the skeleton, using in vitro exposures and assays. Using both the pre-osteoblast and osteoblast we hypothesized that manzamine-A would affect cell viability and function of these important cells. Further, we hypothesized that as Six1 is a predicted mRNA target of the emerging drug manzamine-A, this target would have altered expression in our osteoblast lineage cells.

## 2. Results

**Figure 1:**
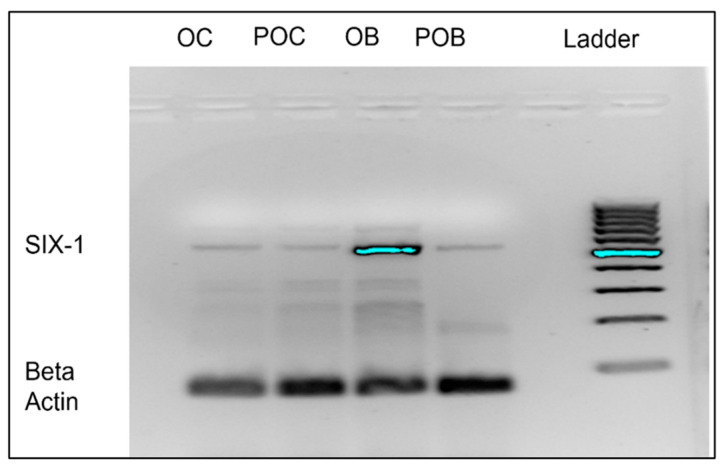
Osteoblast highly expresses Six1 mRNA. Representative (of *n* = 3) agarose gel demonstrating great expression of Six1 target in mature differentiated osteoblasts. OC = Osteoclast; POC = Macrophage; OB = Osteoblasts; POB = Pre-Osteoblasts.

To determine if *Six1* mRNA is expressed in pre-osteoblasts and osteoblasts, agarose gel electrophoresis compared presence of *Six1*. β-actin was used as an endogenous mRNA control and we utilized cells of a different lineage (monocyte/macrophage) cells, raw 264.7 cells as control cells for study purposes. *Six1* was found to be expressed in pre-osteoblasts and particularly enriched expression in osteoblasts as shown in Figure 1. These data further supported our focus on the osteoblast as a potential target of the manzamine-A drug.

**Figure 2:**
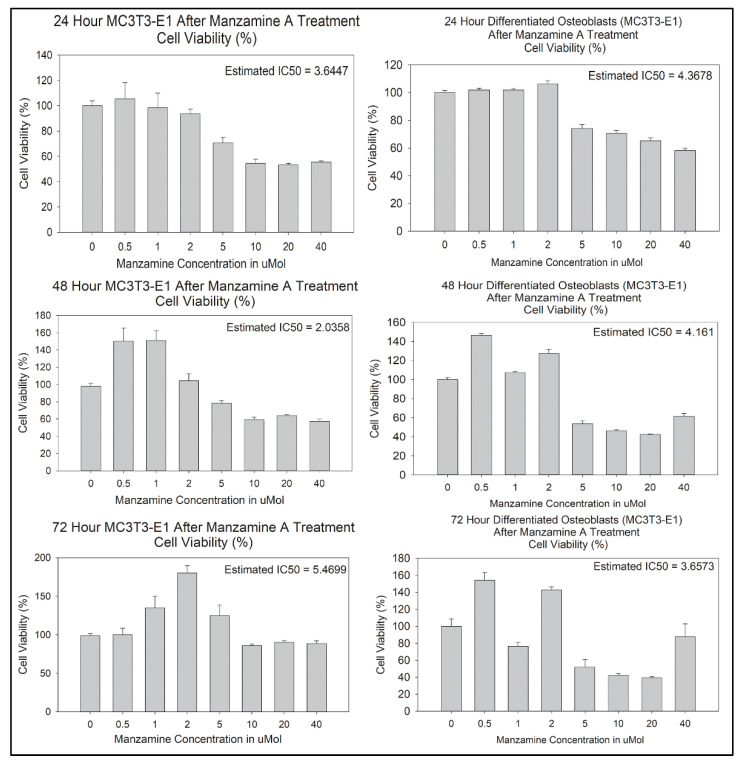
Manzamine treatment alters osteoblast lineage cell viability. MTS assay was used to determine IC50 values for downstream experimental studies. Note alteration to viability at all post-treatment timepoints. (*n* = 3 replicates per treatment).

We next determined IC50 values of manzamine-A in pre-osteoblasts after 24-, 48-, and 72-h of treatment using standard MTS viability assay, Figure 2. The estimated IC50 values were 3.6447 μmol at 24 h, 2.0358 μmol at 48 h, and 5.4699 μmol at 72 h. These assays were repeated in mature osteoblasts after differentiation, Figure 2. Resulting estimated IC50 values were 4.3678 μmol at 24 h, 4.161 μmol at 48 h, and 3.6573 μmol at 72 h. To interrogate additional cellular effects of manzamine A on the osteoblast cell lineage doses of 2.5 μmol and 5 μmol were chosen for further experimentation.

**Figure 3:**
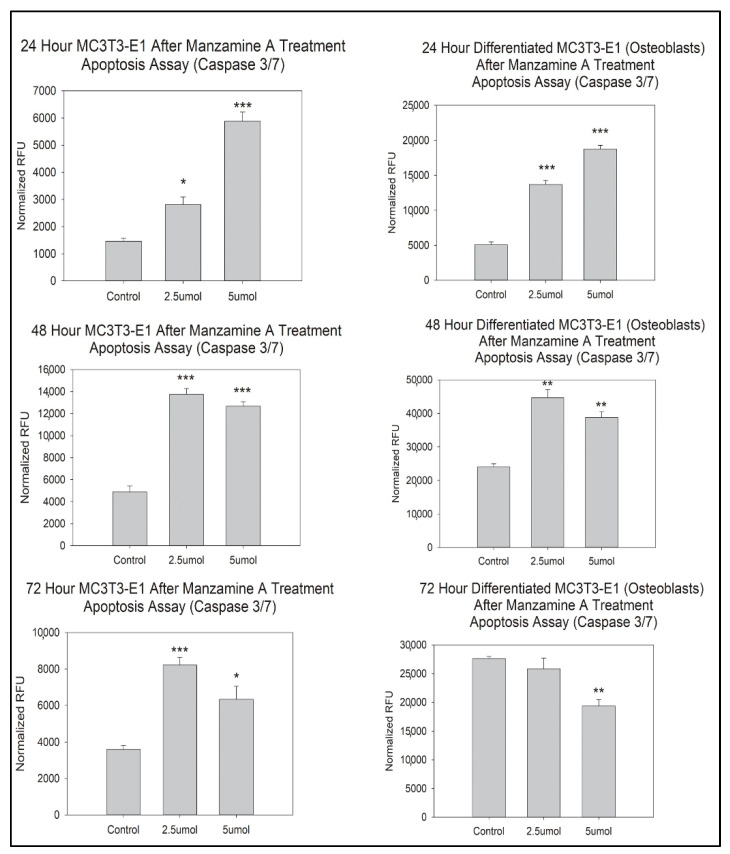
Manzamine treatment drives apoptosis in osteoblast lineage cells. Note increases in apoptosis for most comparisons with the exception of 72 h mature osteoblasts. *n* = 3 replicates per treatment. * *p* < 0.05, ** *p* < 0.01, *** *p* < 0.001. (RFU = Relative Fluorescent Unit).

Caspase 3/7 assay was utilized to determine how manzamine A affected programmed cell death, apoptosis, in our osteoblast cell lines. In our pre-osteoblasts, there was a statistically significant increase in apoptosis at 24 h, 48 h, and 72 h in both the 2.5 μmol and 5 μmol doses groups, Figure 3. This effect was mirrored in our mature osteoblast cells at 24 and 48 h where statistically significant increases in apoptosis were observed, Figure 3. However, at 72 h, there was a statistically significant decrease in cellular apoptosis in the 5 μmol manzamine dosed group. We interpret this as the result of most cellular apoptosis having already robustly occurred in this dosed group. This is supported by our observation of the cells in wells at that time point (Supplemental Figure S1).

**Figure 4:**
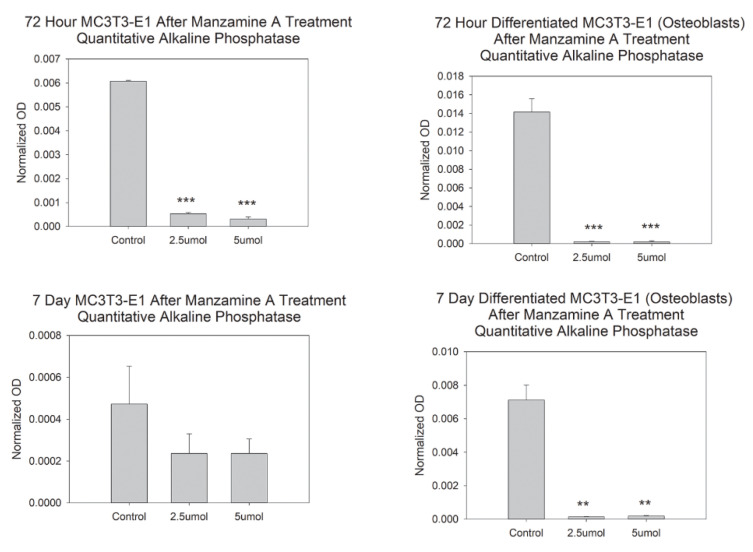
Manzamine decreases osteoblast function. Quantitative alkaline phosphatase activity was decreased by manzamine treatments. *n* = 3 replicates per treatment. ** *p* < 0.01, *** *p* < 0.001. (OD = Optical Density).

To determine the effects of manzamine A on osteoblast lineage function a quantitative alkaline phosphatase assay was used. Our data suggest great and statistically significant decreases in alkaline phosphatase by enzymatic reaction detection in our pre-osteoblasts after 72 h. This effect was not observed at the 7-day timepoint, Figure 4. We attribute the lack of change at 7-days due to the length of the assay in which cells were likely able to renew or stabilize after the dose treatment. Experimentation was repeated in our differentiated osteoblast blast and significant decreases in alkaline phosphatase were observed at both 72 h and at 7 days for both delivered doses. These data support a scenario where manzamine-A is likely to disrupt the function of the osteoblast to produce bone matrix.

**Figure 5:**
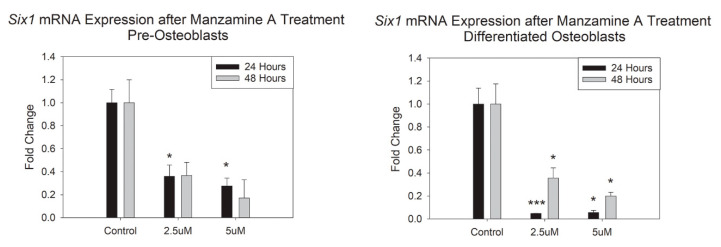
Manzamine treatment results in decreased Six1 mRNA expression. mRNA expression changes represented as fold change. Note significant decreases especially for the mature osteoblast cells. *n* = 3 replicates per treatment. * *p* < 0.05, *** *p* < 0.001. (OD = Optical Density).

We sought to interrogate how manzamine-A treatment would alter Six1 mRNA expression in our cells of the osteoblast lineage. Our data demonstrated marked and significant decreases in mRNA expression after 24 h in our pre-osteoblasts and after 24 and 48 h in our differentiated osteoblasts for both the doses utilized here, Figure 5. These support the interaction between manzamine-A and *Six1* in resulting cell function.

**Figure 6:**
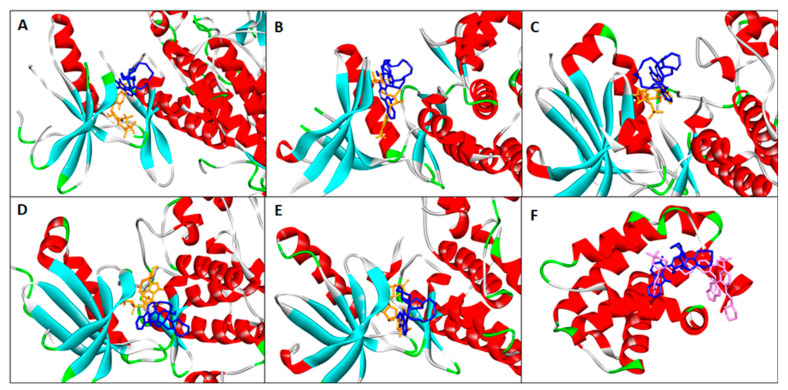
In silico molecular docking of manzamine A to apoptosis regulating proteins. (**A**) Iκb; (**B**) JAK2; (**C**) AKT; (**D**) PKC; (**E**) FAK; and (**F**) Bcl-2. Manzamine A, ATP, and ventoclax are shown as blue, orange, and pink structures, respectively.

Finally, we sought to apply in silico binding experiments to predict manzamine A activity as an inhibitor of cell viability. Protein kinase is an important signaling protein in many biological pathways including cell proliferation and apoptosis. Most of the protein kinase inhibitors bind competitively at the highly conserved ATP-binding domain [93,94,95]. Protein kinase inhibitors have been shown to be active against multiple protein kinases due to the conservation nature of the ATP-binding domain. Manzamine A has been previously shown as an inhibitor of three protein kinases, i.e., GSK3b, CDK5, and RSK1 [96,97]. It is safe to assume that manzamine A may have activity against more protein kinases due to its reported activities for multiple targets and diseases. In this study, manzamine A is docked at the ATP-competitive domain of seven protein kinases (TGF-β, Iκb, JAK2, PI3K, AKT, PKC, and FAK) involved in cell proliferation, survival, and apoptosis pathways. The binding affinities of manzamine A to these proteins were compared with ATP. The results showed that manzamine A has a higher affinity than ATP for Iκb, JAK2, AKT, PKC, and FAK, suggesting its potential as an inhibitor for these proteins (Figure 6 and Table 1). Inhibition of these proteins will result in the downregulation of cell proliferation and survival proteins, and promote expression of caspase 3/7 leading to apoptosis. Manzamine A was also docked to Bcl-2 protein, an important regulator of apoptosis. Inhibition of Bcl-2 will lead to higher expression of caspase 3/7 and apoptosis. Manzamine A showed good binding affinity to Bcl-2 (−10.1 kcal/mol). This indicated manzamine A potential as a Bcl-2 inhibitor although it may be less active than the known Bcl-2 inhibitor venotoclax [98] (binding affinity = −12.2 kcal/mol).

## 3. Discussion

This is the first step into understanding how manzamine-A may affect organ systems that are not the intended target of this emerging drug. Further these experiments provide a platform by which other cell types, and more importantly other emerging natural products can be studied for potential health effects. Overall, our data paints a picture of effects that manzamine-A would have after short term treatment on a bone cell line. Namely, decreases in osteoblast function should be expected and further that bone as an organ is a potential target of these drugs. This implication takes on many forms that necessitate further study as manzamine-A has shown great promise in targeting of disease processes such as cancers. Population indications for manzamine A are widespread as indicated above. Thus, individuals at risk of bone effects due to manzamine A use could include the fetus and or offspring of a dosed expectant mother, an adolescent or young adult in a positive curve for the building of bone density, and more directly those patients at risk of bone wasting diseases such as osteopenia or osteoporosis, the elderly including women after menopause. Other natural products have been shown to target bone related cells, specifically altering bone remodeling via osteoclast activity. Interestingly in this context several drugs have been purported to potentially be useful as a therapeutic where there is a bone wasting disease [99,100,101]. Here, our data suggests manzamine-A will also target bone as an organ but will likely result in further bone loss in stark contrast to Thiaplakortone B or Hymenialdisine for example.

Limitations here include the approach. These initial data were collected using an in vitro modeling system only. Future research will necessitate in vivo dosing of manzamine A likely initially in experimental models to determine the widespread and localized effects in may have on organ systems including bone. Further, a single target was chosen to confirm our bio-informatic conclusion that Six1 was targeted by manzamine-A. This hypothesis proved true, but it is likely that manzamine A has pleiotropic effects including other *Six1* associated mRNA pathway targets (*Gro, Mdfi, Dach,* and *Eya1*) and pathways associated with bone development and health (WNT, NOTCH, PI3K/AKT). Further, although limited in scope, in silico binding experiment has provided a glimpse of possible targets associated with these pathways and supported his hypothesis. Future research will be needed to focus on exploring these and additional molecular targets of the drug to get a clear picture of potential effects in multicellular systems. Furthermore, if manzamines continue to prove to have undesirable side effects such as bone loss, approaches to mitigate these effects will be explored [102]. The next logical target are other bone cells (progenitor cells, osteoclast, osteocytes) as well as re-interrogating the effects of manzamine A on bone remodeling and homeostasis [103] Overall, this initial study did show dramatic effects of manzamine A on the osteoblast, where viability was altered, apoptosis was increased, and function of the cell was diminished.

## 4. Materials and Methods

MC3T3-E1 cells (Subclone 4 CRL-2593) were obtained from ATCC (Manassas, VA, USA) and maintained as recommended to produce pre-osteoblast cells for studies. Briefly, cells were maintained in a 75 cm^2^ flask using Alpha Eagle Minimum Essential Medium (Alpha-MEM, Lonza, Walkersville Inc., Walkersville, MD, USA, BE02–002F) with 10% Fetal Bovine Serum (FBS, Atlanta Biologicals, Atlanta, GA, USA, S11150H) and 1% Penicillin/Streptomycin (Lonza, 10k/10k 17–602E) until 85% confluent when they were moved to a 175 cm^2^ flask after trypsin-EDTA (0.1%, Gibco/Fisher Scientific, Hampton, NH, USA, 15400–054) dissociation. Cells (Pre-osteoblasts) were subcultured as necessary throughout the experimentation. To induce osteoblast differentiation 0.25 mM ascorbic acid (Fisher Scientific, Hampton, NH, USA, A61–25), 0.1 μm dexamethasone (Fisher Scientific, Hampton, NH, USA, AC230300010), and 10 mM β-glycerophosphate (Fisher Scientific, Hampton, NH, USA, L03425) were added to standard Alpha-MEM media to produce osteogenic media (OM, Osteoblasts). Control cells for PCR study of Six1 mRNA expression were inclusive of Raw 264.7 cells (ATCC, Manassas, VA, USA, TIB-71) maintained as macrophage precursors or differentiated to mature osteoclast phenotype. These cells were maintained in a 75 cm^2^ flask using Dulbecco’s Modified Eagle’s Medium (DMEM, Lonza, Basel, Switzerland, 12604F) with 10% FBS and 1% Penicillin/Streptomycin until 85% confluent, when they were moved to a 175 cm^2^ flask after cell scraper dissociation. Cells were sub-cultured as necessary throughout the experimentation. To induce mature osteoclast phenotype murine RANK Ligand 50 ng/mL (Peprotech, Secaucus, NJ, USA, 315–11) was added to the standard DMEM media (RANKL, Osteoclasts).

Manzamine-A was produced by co-author Dr. Mark Hamann’s laboratory at Medical University of South Carolina following previously published protocols. Briefly, lipophilic alkaloid extracts were previously prepared from the Indonesian sponge *Acanthostrongylophora* (collected from Manado Bay, Northern Sulawesi, Indonesia, in 2003). Crude material was separated using vacuum liquid chromatography, and manzamine was purified by crystallization. Purified manzamine was then transformed into its hydrochloride salt and recrystallized to reach high purity (>99%) and optimized aqueous solubility as described previously [41,44,45].

Pre-osteoblasts, mature osteoblasts, macrophages, and osteoclasts, were seeded in triplicate at a density of 300,000 cells per well in triplicate in 6 well culture plates and treated with control un-supplemented media, and appropriate differentiation media as defined above. After 24 h in isolated culture, RNA was isolated using the OMEGA bio-tek E.Z.N.A. Total RNA kit 1 (Omega Bio-tek, Norcross, GA, USA, R6834-02) according to manufacturer’s protocol. Quality and quantity of RNA was assessed using a Synergy Hi Microplate reader and a Take3 Microvolume Plate (BioTek, Winooski, VT, USA). Complimentary DNA synthesis was performed using Quanta qScript cDNA Synthesis reagents following manufacturers protocol (Quanta Biosciences, Beverly, MA, USA, 95047-025). Presence of neurotransmitters and GPCRs was determined via PCR using cDNA, designed primers from Integrated DNA Technologies (Coralville, IA, USA,) (Table 1), Platinum Taq DNA Polymerase (Fisher Scientific, Hampton, NH, USA, 100021273), and separation on 1.5% agarose gels employing primers for *Six1* (GCTGTCACC GGGCCTATTTA/Reverse ATGAGCAAGCCAACCCTGTT) *β-Actin* (Forward GCAGGAGTACGATGAGTCCG/Reverse ACGCAGCTCAGTAACAGTCC) as a control. Annealing temperature was optimized to 53C. Each assay was repeated three independent times.

Cell viability was measured using a CellTiter 96^®^ AQ_ueous_ One Solution Cell Proliferation Assay (MTS, Promega, Madison, WI, USA). The conversion of MTS tetrazolium into Formazan is directly proportional to the number of viable cells in each well. Osteoblast lineage cells were seeded in 96 well plates at a density of 4000 cells/well and treated with Manzamine at serial concentrations (0.5–40 µMol) compared to a control group (0 umol) without exposure to Manzamine and then assayed at 24, 48, and 72 h (triplicate replicates). Inhibitory Concentration (IC50) values were determined using AAT BioQuest Graph and used for all downstream studies.

Cellular apoptosis levels were measured using Apo-ONE Homogenous Caspase 3/7 Assay (Promega, Madison, WI, USA). This assay measures the amount of Caspase 3 and Caspase 7 activity to quantify apoptosis levels. Cells were seeded in 96 well plates at a density of 4000 cells/well and treated with Manzamine at 2.5 µmol and 5 µmol concentrations compared to a control group without exposure to Manzamine then assayed at 24, 48, and 72 h (triplicate replicates) On assay, cells were incubated with 100 μL/well substrate/buffer solution (1:100). Contents were mixed for 30 s at 300 rpm and incubated at room temperature for one hour. Fluorescence was measured using a 96-well plate reader (Biotek, Winooski, VT, USA) with excitation at 485 and emission at 530 nm.

Functional levels of pre-osteoblasts and osteoblasts were measured using a SIGMA*FAST* p-nitrophenyl phosphate alkaline phosphatase assay from Sigma-Aldrich (St. Louis, MO, USA). This assay measures the quantification of the reaction of the enzyme, alkaline phosphatase, an important factor that leads to mineralization of bone. Cells were seeded in 96 well plates at a density of 4000 cells/well and treated with Manzamine at 2.5 μmol and 5 μmol concentrations compared to a control group without exposure to Manzamine then assayed at 24, 48, and 72 h (triplicate replicates). On assay medium was removed from cells, and cell lysis was performed using Triton ×100 at 0.01% (Sigma). After 30 min of incubation at 4 °C, deionized water and a p-Nitrophenyl phosphate solution were added to the lysis buffer. Three control wells containing no cells were also treated and served as blank controls to mathematically subtract the effects of the lysis buffer and water on final optical densities. Plates were incubated at room temperature in the dark for 30 min. The absorbance at 405 nm was recorded with a 96-well plate reader (Biotek, Winooski, VT, USA). ALP activity was then calculated using the following formula: ((optical density—the mean optical density of the control wells) × total volume × dilution)/(18.45 × sample volume).

To quantify expression the specific mRNA target *SIX1*, cDNA was subjected to quantitative PCR using Applied Biosystems TaqMan Gene Expression Master Mix and targeted TaqMan gene expression assay (Mm00808212_m1) and normalized to control endogenous *Gapdh* activity (Mm99999915_g1). Briefly cells were cultured in 6 well plates with a seeding density of 300,000 cells/well. Pre-osteoblasts and osteoblasts were treated with 2.5 μmol and 5 μmol doses of Manzamine compared to a control group without exposure to Manzamine. Cells were then harvested at 24 and 48 h for RTQPCR experiments (triplicate replicates). RNA was isolated from both cell types using the RNA Isolation protocol from the E.Z.N.A. Total RNA Kit from Omega. After RNA Isolation, the RNA was transcribed into complementary cDNA using the cDNA Synthesis protocol from qScript cDNA Synthesis Kit from QuantaBio. cDNA is then used as the template strand in a quantitative PCR reaction using a TaqMan Fast Advanced Master Mix. Data were normalized to GAPDH RNA expression by ΔCT. Quantitative data were compared for gene expression changes due to treatment with manzamine by ΔΔCT methodology. Previously published statistical analysis methodology was used to determine differences for gene expression after manzamine related the target of interest [104]. Differences were considered significant if *p* ≤ 0.05.

All statistical analyses were done by comparing the Manzamine dosed groups to the control groups using a standard t test or non-parametric Mann–Whitney U after assessing normality with a Shapiro–Wilk test and homogeneity of variance with a Levene’s test using Welch’s correction for variance when needed. Significance was determined if *p* values were less than 0.05.

The structures of manzamine A, ATP, and venetoclax were optimized using the MM2 energy-minimized function in the Chem3D Ultra version 16.0. The crystal structures of the receptor proteins (Table 1) were obtained from the Protein Data Bank [105,106]. AutoDockTools version 1.5.6 were used to prepare the receptor proteins and ligands for the molecular docking experiment. The grid box parameters used were: grid box spacing = 1.0 Å; x-dimension = y-dimension = z-dimension = 20. AutoDock Vina program was used to perform the docking and calculate the binding affinity [107,108]. The results were processed and analyzed using the BIOVIA Discovery Studio Visualizer version 17.2.0 (Dassault Systèmes, San Diego, CA, USA). The binding affinities are summarized in Table 1.

## Figures and Tables

**Table 1:** Binding affinity (kcal/mol) and coordinates.

	TGF-b	Ikb	JAK2	PI3K	AKT	PKC	FAK	Bcl-2
**PDB ID**	6B8Y	4KIK	6WTO	4FA6	3MVH	1XJD	3BZ3	6O0K
**x-centre**	5.788	49.323	−21.148	44.555	24.812	56.783	10.234	−14.226
**y-centre**	9.372	30.567	−14.051	13.306	5.644	8.908	2.763	1.146
**z-centre**	5.017	−56.867	8.259	31.313	18.343	2.494	5.109	−10.800
**Manzamine A**	10.3	−8.2	−10.8	−6.6	−9.2	−10.3	−9	−10.1
**ATP**	−8.2	−7.7	−8	−7	−7.8	−7.3	−7.7	n.a.
**Venetoclax**	n.a.	n.a.	n.a.	n.a.	n.a.	n.a.	n.a.	−12.2

n.a.—not applicable.

## Data Availability

Raw data provided by request to corresponding author.

## Supplementary Materials

The following supporting information can be downloaded at: https://www.mdpi.com/article/10.3390/md20100647/s1, Figure S1: Phase Images of Cells Treated with Manzamine.

## Abbreviations

ALP	Alkaline Phosphatase
°C	Degrees Celsius
COVID	Corona Virus Disease
Dach	Dachshund Homolog 1
DNA	Deoxyribonucleic acid
Eya1	Eyes absent homolog 1
Gro	Groucho
HIV	Human Immunodeficiency Virus
IC50	Inhibitory concentration 50%
Mdfi	MyoD Family Inhibitor
mRNA	Message ribonucleic acid
MTS	3-(4,5-dimethylthiazol-2-yl)-5-(3-carboxymethoxyphenyl)-2-(4-sulfophenyl)-2H-tetrazolium
NIH	United States National Institutes of Health
Notch	Notch Receptor
PCR	Polymerase Chain Reaction
qrtPCR	Quantitative Real Time Polymerase Chain Reaction
PI3K/AKT	Phosphoinositide 3-kinases/Protein kinase B
RNA	Ribonucleic Acid
Six1	Sine Oculis Homeobox
US-CDC	United State Center for Disease Control
WNT	Wingless Intergrated
