# Supplementary figures and images for: Manzamine-A Alters In Vitro Calvarial Osteoblast Function
Source: Mar Drugs. 2022 Oct 19;20(10):647. doi: 10.3390/md20100647 (PMC9604769; doi:10.3390/md20100647)

Control

2.5  $\mu\text{mol}$

5  $\mu\text{mol}$

72 hrs

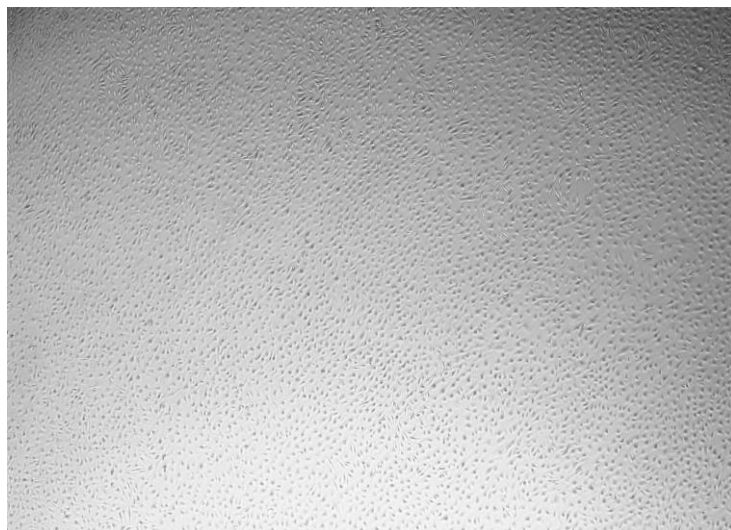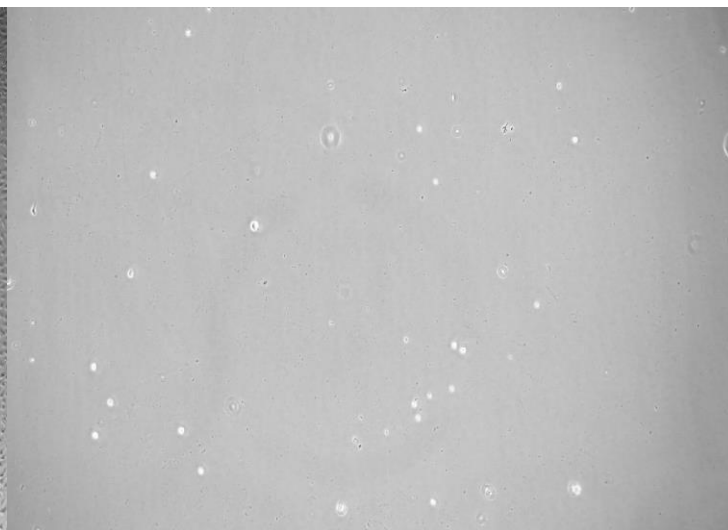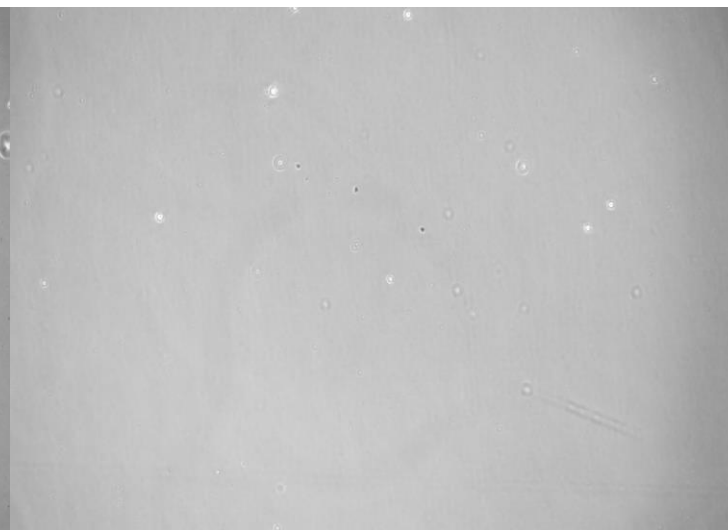

7 days

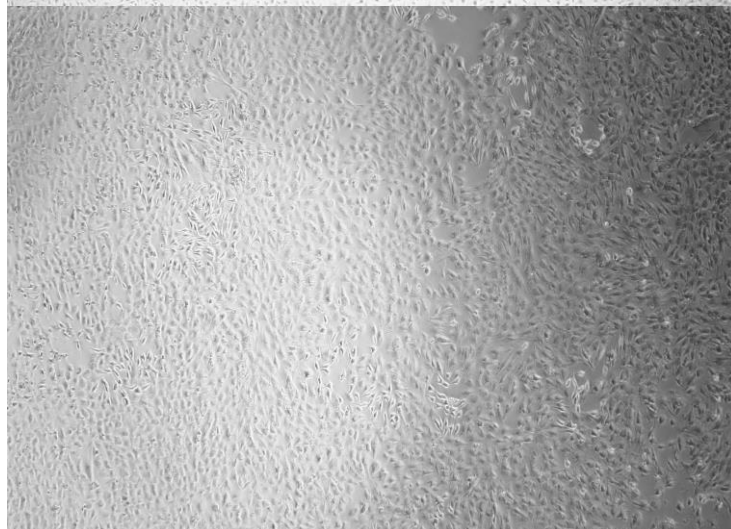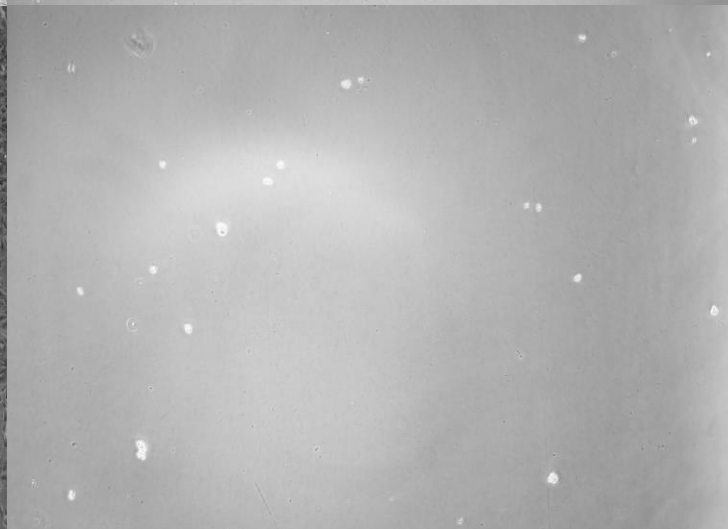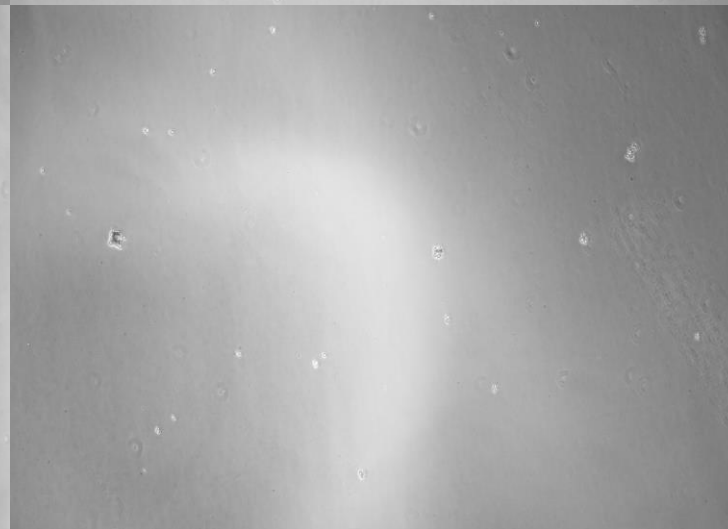

Supplement: Supplementary file 1 [file marinedrugs-20-00647-s001.zip › marinedrugs-1941321-supplementary.pdf]
